# Stroke Prevention in Atrial Fibrillation: A Scientific Statement of *JACC: Asia* (Part 1)

**DOI:** 10.1016/j.jacasi.2022.05.005

**Published:** 2022-08-16

**Authors:** Chern-En Chiang, Tze-Fan Chao, Eue-Keun Choi, Toon Wei Lim, Rungroj Krittayaphong, Mingfang Li, Minglong Chen, Yutao Guo, Ken Okumura, Gregory Y.H. Lip

**Affiliations:** a General Clinical Research Center, Taipei Veterans General Hospital, Taipei, Taiwan; b Division of Cardiology, Department of Medicine, Taipei Veterans General Hospital, Taipei, Taiwan; c School of Medicine, National Yang Ming Chiao Tung University, Taipei, Taiwan; d Institute of Clinical Medicine, and Cardiovascular Research Center, National Yang Ming Chiao Tung University, Taipei, Taiwan; e Department of Internal Medicine, Seoul National University Hospital, Seoul, Republic of Korea; f National University Heart Centre, National University Hospital, Singapore, Singapore; g Division of Cardiology, Department of Medicine, Faculty of Medicine Siriraj Hospital, Mahidol University, Bangkok, Thailand; h Division of Cardiology, the First Affiliated Hospital of Nanjing Medical University, Nanjing, China; i Department of Pulmonary Vessel and Thrombotic Disease, Sixth Medical Centre, Chinese PLA General Hospital, Beijing, China; j Liverpool Centre for Cardiovascular Science, University of Liverpool & Liverpool Heart and Chest Hospital, Liverpool, United Kingdom; k Division of Cardiology, Saiseikai Kumamoto Hospital, Kumamoto, Japan; l Department of Clinical Medicine, Aalborg University, Aalborg, Denmark

**Keywords:** Asia, atrial fibrillation, non–vitamin K antagonist oral anticoagulant, stroke, vitamin K antagonist, ABC, atrial fibrillation better care, AF, atrial fibrillation, NOAC, non-vitamin K antagonist oral anticoagulant, OAC, oral anticoagulant, PCI, percutaneous coronary intervention, TAVI, transcatheter aortic valve implantation, VKA, vitamin K antagonist

## Abstract

Atrial fibrillation is the most common sustained cardiac arrhythmia and is associated with substantial increases in the risk of stroke and systemic thromboembolism. With the successful introduction of the first non-vitamin K antagonist direct oral anticoagulant (NOAC) in 2009, the role of vitamin K antagonists has been replaced in most clinical settings except in a few conditions when NOACs are contraindicated. Data for the use of NOACs in different clinical scenarios have been accumulating in the recent decade, and a more sophisticated strategy for atrial fibrillation patients is now warranted. *JACC: Asia* recently appointed a working group to summarize the most updated information regarding stroke prevention in AF. This statement aimed to provide possible treatment option in daily practice. Local availability, cost, and patient comorbidities should also be considered. Final decisions may still need to be individualized and based on clinicians’ discretion. This is the part 1 of the whole statement.

Stroke and systemic thromboembolism are the most clinically important complications observed in patients with atrial fibrillation (AF). Overall, the incidence of stroke in patients with AF is 4- to 5-fold higher than that in patients without AF.^1^ AF may be asymptomatic, but confers a poor prognosis if undetected and treated, especially post stroke.^2^ The complexity of AF requires multifaceted, holistic, and multidisciplinary approaches to the management of AF patients.^3^ Stroke prevention is arguably the single most important strategy among these.

With the successful introduction of the first non–vitamin K antagonist oral anticoagulant (NOAC) in 2009, the role of vitamin K antagonists (VKAs) has been replaced in most clinical settings except in a few conditions when NOACs are contraindicated.^4^^,^^5^ Data for the use of NOACs in different clinical scenarios have been accumulating in the recent decade, and a more sophisticated strategy for AF patients is now warranted.

*JACC: Asia* recently convened a working group to summarize most updated information regarding stroke prevention in AF, with a focus on Asia. Details of working group members are shown in the Supplemental Table 1. The working group carefully reviewed the most recent data and formulated an updated statement for stroke prevention in AF patients, especially focusing on prioritizing specific NOACs in different clinical settings.

## Prevalence of Af in Asia

In a recent meta-analysis of 58 articles from 8 countries in Asia, the community- and hospital-based AF prevalence ranged from 0.37% to 3.56% and 2.8% to 15.8%, respectively.^6^ The prevalence rate of AF is continuously increasing in Asia as in Western countries; for example, the prevalence rate of AF in Taiwan was around 1.5% in year 2020 and that will reach 4.0% in year 2050.^7^

**Figure 1:**
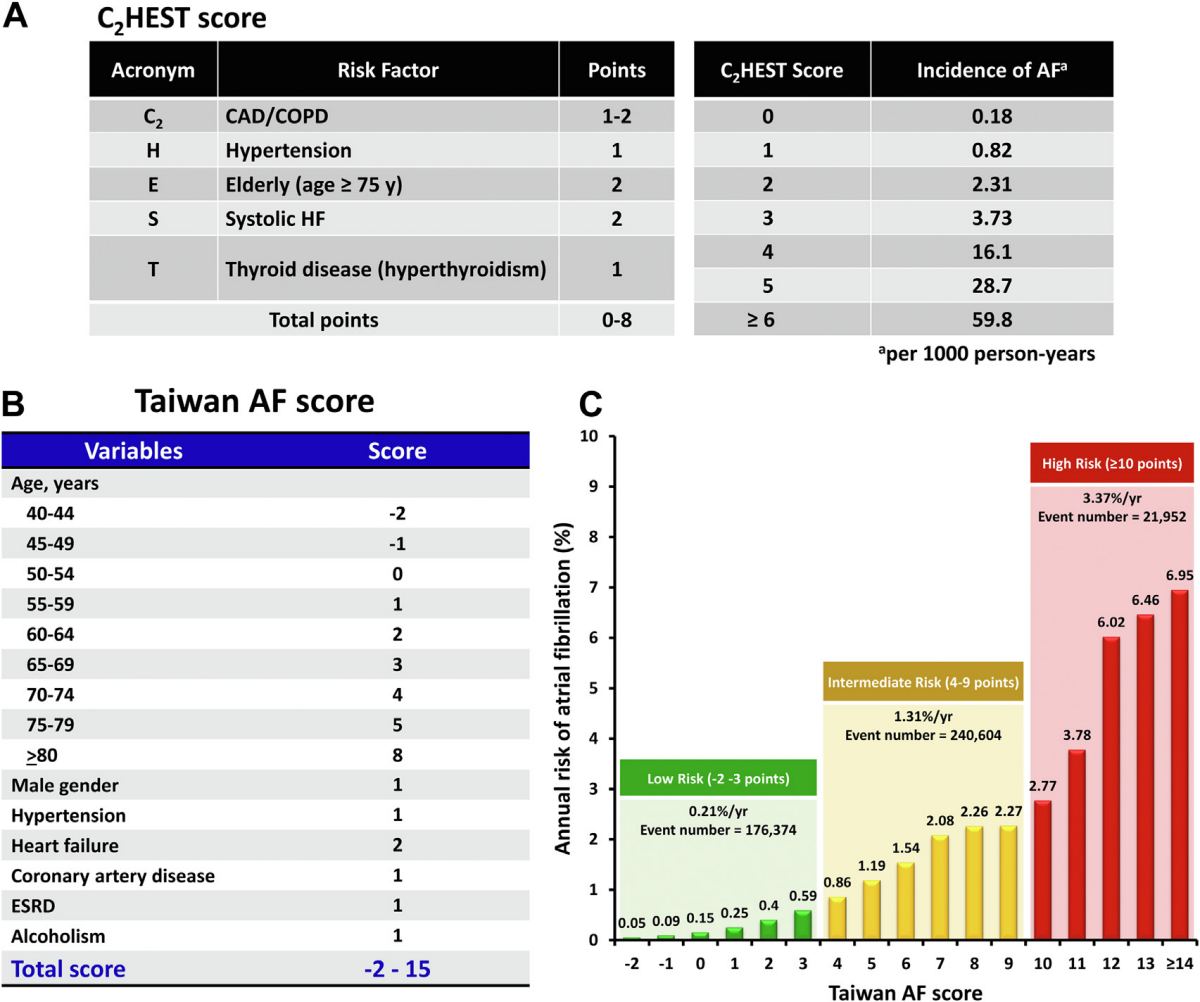
C_2_HEST and Taiwan AF Scores for Prediction of Incident AF C_2_HEST and Taiwan atrial fibrillation (AF) scores can predict the risk of incident AF. **(A)** The calculation tables of the C_2_HEST score and incidence of AF (per 1,000 person-years). **(B)** The calculation table of the Taiwan AF score. **(C)** The annual risk of AF based on the Taiwan AF score. Adapted with permission.^8^^,^^9^ C_2_HEST = coronary artery disease or chronic obstructive pulmonary disease (1 point each); hypertension (1 point); elderly (age ≥75 years, 2 points); systolic heart failure (2 points); thyroid disease (hyperthyroidism), 1 point); CAD = coronary artery disease; COPD = chronic obstructive pulmonary disease; ESRD = end-stage renal disease; HF = heart failure.

Recently, 2 schemes have been developed for the prediction of incident AF for patients in Asia and Taiwan, namely, the C_2_HEST (coronary artery disease or chronic obstructive pulmonary disease [1 point each]; hypertension [1 point]; elderly [age ≥75 years, 2 points]; systolic heart failure [2 points]; thyroid disease [hyperthyroidism], 1 point) score and Taiwan AF score, respectively.^8^^,^^9^ The C_2_HEST score was derived from 471,446 Chinese subjects with 921 incident AF cases, and validated in the Korean nationwide data, whereas the Taiwan AF score was derived from 7,220,654 Taiwan patients with 438,930 incident AF cases.^8^^,^^9^ The area under the curve for the prediction of AF was 0.749 (95% CI: 0.729-0.769) for the simple C_2_HEST score and 0.756 (95% CI: 0.755-0.757) for the Taiwan AF score.^8^^,^^9^ The calculation rules of these 2 scoring schemes and the risk of incident AF in different score strata are shown in Figure 1.

### Consensus statement

• The prevalence of AF is increasing in Asia, and several scoring schemes (such as C_2_HEST and Taiwan AF scores) can be used to predict the risk of incident AF for Asian patients.

## Assessment/re-Assessment of Stroke and Bleeding Risks

For AF patients in Asia, the annual risk of ischemic stroke was approximately 3.0% (range: 1.60%-4.95%) based on a pooled analysis of 8 studies.^6^ Most international guidelines recommends use of the CHA_2_DS_2_- score and the HAS-BLED score to assess the stroke and bleeding risks of AF patients respectively.^5^^,^^10^, ^11^, ^12^ The HAS-BLED score has also been validated in European and Asian AF patients taking NOACs, and draws attention to the modifiable bleeding risk factors (unlike other scores) and facilitates identification of high bleeding risk patients for early review and follow-up.^13^ The usefulness of the CHA_2_DS_2_-VASc and HAS-BLED scores in the prediction of ischemic and bleeding events has been well validated for AF patients in Asia.^14^

**Figure 2:**
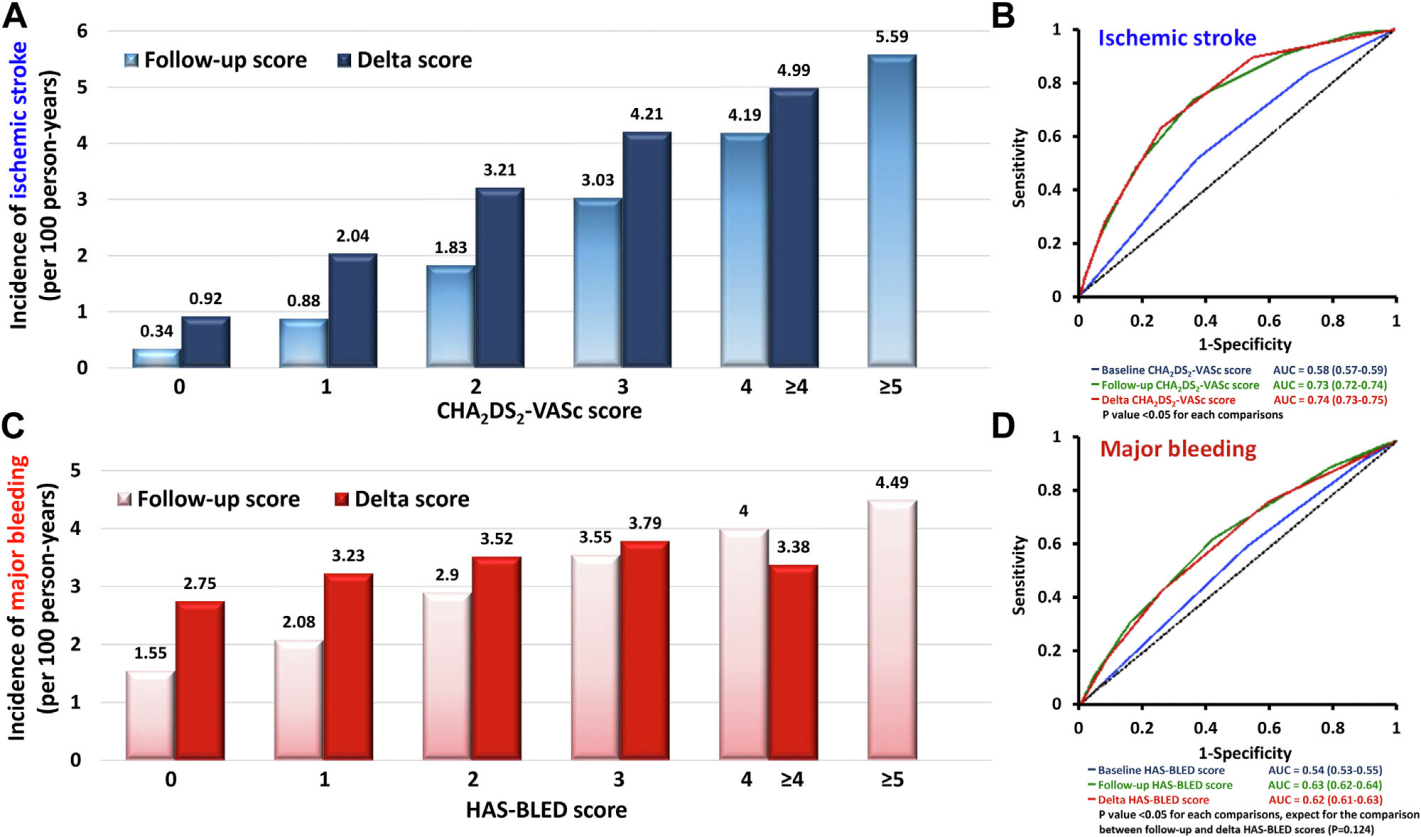
Dynamic Natures of CHA_2_DS_2_-VASc and HAS-BLED Scores The stroke and bleeding risks of AF patients were not static; patients would become older and acquired incident comorbidities. **(A)** The incidence rate of ischemic stroke according to the follow-up CHA_2_DS_2_-VASc scores and delta-CHA_2_DS_2_-VASc scores (the difference between the baseline and follow-up scores). **(B)** The area under the receiver-operating characteristic curves (AUCs) for the baseline, follow-up, and delta-CHA2DS2-VASc scores in predicting ischemic stroke. **(C)** The incidence rate of major bleeding according to the follow-up HAS-BLED scores and delta-HAS-BLED scores (the difference between the baseline and follow-up scores). **(D)** The AUCs for the baseline, follow-up, and delta-HAS-BLED scores in predicting major bleeding. Adapted with permission from Chao et al.^15^^,^^16^ AUC = area under curve; CHA_2_DS_2_-VASc = congestive heart failure, hypertension, age ≥75 (doubled), diabetes, stroke (doubled)-vascular disease, age 65-74 and sex category (female); HAS-BLED = hypertension, abnormal renal/liver function (1 point each), stroke, bleeding history or predisposition, labile INR, elderly (> 65 years), drugs/alcohol concomitantly (1 point each).

It is important to understand that the stroke and bleeding risks of AF patients were not static, as patients will become older and acquire incident comorbidities.^15^ For example, in a study from Taiwan that enrolled 14,606 incident AF patients with a CHA_2_DS_2_-VASc score of 0 (males) or 1 (females) at baseline, approximately 16.1% of men and 16.2% of women had CHA_2_DS_2_-VASc score of at least 1 (men) or 2 (women) at 1 year after incident AF.^16^ Both follow-up CHA_2_DS_2_-VASc scores and delta-CHA_2_DS_2_-VASc scores (the difference between the baseline and follow-up scores) were associated with a higher risk of ischemic stroke, and had better predictive values compared to the baseline CHA_2_DS_2_-VASc score (Figure 2).^16^ Importantly, the prescription of oral anticoagulants (OACs) when patients’ CHA_2_DS_2_-VASc scores increased was associated with lower risk of clinical events.^17^ More recently, machine-learning models that accounted for dynamic changes in risk including newly acquired risk factors improved the prediction of AF-associated stroke.^18^ The details of machine learning will be discussed in part 2 of the statement.

Bleeding risks as assessed by the HAS-BLED score are also dynamic, altered by aging and incident comorbidities (and the mitigation of modifiable bleeding risk factors). The accuracy of the follow-up HAS-BLED score or delta-HAS-BLED scores in the prediction of major bleeding was significantly higher than that of the baseline HAS-BLED score (Figure 2).^15^ In 24,990 anticoagulated AF patients with initial HAS-BLED score ≤2, 5,229 (20.9%) patients had an increment of their HAS-BLED scores to ≥3 at the end of 1 year, mainly due to newly diagnosed hypertension, stroke, bleeding, and concomitant drug therapies.^19^ Given the stroke and bleeding risks of AF patients change over time, the CHA_2_DS_2_-VASc and HAS-BLED scores should be re-assessed regularly, ideally at every patient-physician contact.

There were only few recommendations from guidelines regarding the frequency of risk re-assessment.^12^ For AF patients who acquired incident comorbidities and experienced ischemic stroke, a recent report showed that the interval from the acquirement of incident comorbidities to the occurrence of ischemic stroke was only 4.4 months in 90% of patients.^17^ Accordingly, the 2021 Asia Pacific Heart Rhythm Society (APHRS) consensus suggested the stroke risk of AF patients should be re-assessed regularly (at least annually and every 4 months if possible).^5^

It should be strongly emphasized that a high HAS-BLED score should not be a reason for not prescribing or withholding OACs for AF patients. For AF patients in Asia who had only 1 nongender stroke risk factor but a high bleeding risk (HAS-BLED score ≥3), the use of OACs was associated with a lower risk of composite adverse events of ischemic stroke, intracranial hemorrhage, or mortality (4.19/100 person-years vs 5.22/100 person-years, adjusted HR: 0.781, *P* = 0.04).^20^ For anticoagulated AF patients who had a baseline HAS-BLED score of 0-2 that increased to ≥3, the continuation of OACs was associated with better clinical outcomes.^19^ The appropriate use of the HAS-BLED score has been tested in the prospective mAFA-II (mobile atrial fibrillation application) II trial.^21^ The use of CHA_2_DS_2_-VASc and HAS-BLED scores for stroke and bleeding risk assessment/re-assessment for AF patients in Asia is summarized in Supplemental Figure 1.

### Consensus statements

• For AF patients in Asia, we recommend CHA_2_DS_2_-VASc and HAS-BLED scores to assess the stroke and bleeding risks, respectively.
• OACs should be provided for AF patients in Asia who have CHA_2_DS_2_-VASc score of ≥1 for males and ≥2 for females.
• Both the stroke and bleeding risks of AF patients are not static, and the CHA_2_DS_2_-VASc and HAS-BLED scores should be assessed regularly at an interval of 4 months to 1 year.
• A high bleeding risk is not the reason for not prescribing or withholding OACs, but it can help physicians to identify and correct modifiable risk factors for bleeding for AF patients who are anticoagulated.

## ABC Pathway

Although substantial focus has been on stroke prevention, a more holistic and integrated approach to AF management has been proposed to improve clinical outcomes in patients with AF.^3^ Stroke only accounts for 1 in 10 deaths related to AF, whereas cardiovascular mortality accounts for approximately 7 in 10 deaths.^22^ To streamline decision-making for a holistic approach to AF management in an integrated manner, the use of the ABC (atrial fibrillation better care) pathway is recommended (Central Illustration).^3^

**Central Illustration:**
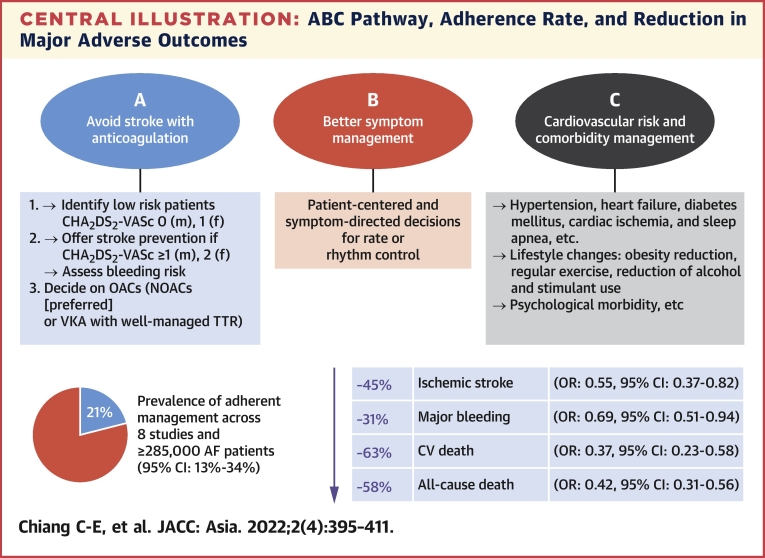
ABC Pathway, Adherence Rate, and Reduction in Major Adverse Outcomes The detailed content the ABC pathway is shown here. According to a recent meta-analysis of 8 studies (≥285,000 patients), a pooled prevalence of ABC-adherent management is only 21%.^23^ Patients treated according to the ABC pathway showed a lower risk of stroke (OR: 0.55; 95% CI: 0.37-0.82), major bleeding (OR: 0.69; 95% CI: 0.51-0.94), cardiovascular death (OR: 0.37; 95% CI: 0.23-0.58), and all-cause death (OR: 0.42; 95% CI: 0.31-0.56). AF = atrial fibrillation; CV = cardiovascular; F = female; m = male; NOAC = non-vitamin K antagonist oral anticoagulant; OAC = oral anticoagulant; TTR = time in therapeutic range; VKA = vitamin-K antagonist.

A systematic review and meta-analysis showed a lower risk of all-cause death (odds ratio [OR]: 0.42; 95% CI: 0.31-0.56), cardiovascular death (OR: 0.37; 95% CI: 0.23-0.58), stroke (OR: 0.55; 95% CI: 0.37-0.82), and major bleeding (OR: 0.69; 95% CI: 0.51-0.94), with management adherent to the ABC pathway compared to noncompliance.^23^ A prospective cluster randomized trial (mAFA-II) showed that patients allocated to the ABC pathway intervention (using mobile health [mHealth] technology) was associated with lower rates of the composite outcome of “ischemic stroke/systemic thromboembolism, death, and rehospitalization” compared with usual care (1.9% vs 6.0%; HR: 0.39; 95% CI: 0.22-0.67; *P* < 0.001).^24^ Rates of rehospitalization were lower with intervention (1.2% vs 4.5%; HR: 0.32; 95% CI: 0.17-0.60; *P* < 0.001).

The improved outcomes with ABC pathway adherence are clearly evident in many studies, including those from Asia.^25^^,^^26^ The ABC pathway intervention also leads to reduced major bleeds and increased oral anticoagulation uptake vs usual care.^21^ The ABC pathway provides a simple management pathway that bridged primary-secondary, and can be understood by everyone: general practitioners, non-cardiologist hospital practitioners, and cardiologists, as well as by patients. The ABC pathway components also serve as a checklist for general practitioners and hospital specialists to discuss with patients.

### Consensus statements

• An integrated care or holistic management approach, based on the ABC pathway, is recommended to improve outcome in the Asian AF population:

A = Avoid stroke with anticoagulation, that is, well-managed warfarin (time-in-therapeutic-range >65% to 70%) or NOAC.

B = Better symptom management with patient-centered symptom-directed decisions for rate or rhythm control.

C = Cardiovascular risk and comorbidity management (blood pressure control, heart failure, cardiac ischemia, sleep apnea, etc) as well as lifestyle changes (obesity reduction, regular exercise, reducing alcohol/stimulants, psychological morbidity, etc).

## Participation of Asian Countries in Clinical Trials and Studies

The 4 Asian subanalyses of NOAC trials reported data from 10 Asian countries/territories: China, Japan, South Korea, Taiwan, Hong Kong, Philippines, Singapore, Malaysia, Thailand, and India.^27^, ^28^, ^29^, ^30^ Details of involvement of Asian countries are shown in Table 1. Real-world studies were mainly from East Asia, including China, Japan, South Korea, and Taiwan. The statements from this consensus can, therefore, be applied in patients living in East Asia and South-East Asia. The data from South Asia were insufficient.

**Table 1:** Asian Countries/Territories Included in Asian Subanalyses of Major NOAC Trials

First Author	Trial Name (N)	Drug	Countries/Territories Included
Hori et al^27^	RE-LY (2,782)	Dabigatran	China, Japan, South Korea, Taiwan, Hong Kong, Philippines, Singapore, Malaysia, Thailand, India
Wong et al^28^	ROCKET AF (932)	Rivaroxaban	China, South Korea, Taiwan, Hong Kong
Goto et al^29^	ARISTOTLE (1,993)	Apixaban	China, Japan, South Korea, Taiwan, Hong Kong, Philippines, Singapore, Malaysia
Yamashita et al^30^	ENGAGE-AF (1,943)	Edoxaban	China, Japan, South Korea, Taiwan

ARISTOTLE = Apixaban for Reduction in Stroke and Other Thromboembolic Events in Atrial Fibrillation; ENGAGE-AF = Effective Anticoagulation with Factor Xa Next Generation in Atrial Fibrillation; RE-LY = Randomized Evaluation of Long-Term Anticoagulation Therapy; ROCKET AF = Rivaroxaban Once Daily Oral Direct Factor Xa Inhibition Compared with Vitamin K Antagonism for Prevention of Stroke and Embolism Trial in Atrial Fibrillation.

## Role of Warfarin

Warfarin is effective in stroke prevention when compared with placebo in Western patients.^31^ The evidence for warfarin in Asian patients is scarce. An optimal international normalized ratio (INR) of 2.0 to 3.0 is more difficult to achieve for Asian patients, possibly because of the differences in polymorphism of the P450 cytochrome CYP2C9 and in the gene for vitamin K epoxide reductase complex 1 (VKORC1).^32^, ^33^, ^34^ In the 4 NOAC trials, Asian patients are prone to bleeding from warfarin use despite a lower INR obtained in trials.^35^

Based on the data from the 4 NOAC trials, we now have ample evidence to replace warfarin with NOACs in stroke prevention for AF.^4^^,^^36^, ^37^, ^38^ The effect sizes of both efficacy and safety of NOAC vs warfarin are greater in Asians vs non-Asians.^39^ Therefore, NOACs should be preferred medications for the stroke prevention in AF for Asian patients, except for a few conditions when NOACs are contraindicated.^12^^,^^40^ However, well-controlled warfarin with adequate time-in-therapeutic range (>65% to 70%) may still be an option in some Asian patients when NOACs are not affordable.

### Consensus statements

• Warfarin should not be the first-line therapy for stroke prevention in AF except when NOACs are contraindicated.
• Well-controlled warfarin with adequate time-in-therapeutic range (>65% to 70%) may still be an option in some Asian patients when NOACs are not affordable.

## Pharmacokinetics and Drug-Drug Interaction of NOACs

Although NOACs have fewer drug-drug interactions compared to traditional OACs, it is still essential to be mindful of them when prescribing to patients who may be on many other medications, including some commonly prescribed for patient with AF. There are numerous online resources to check for such interactions, and we have summarized the common ones as well as the pharmacokinetic profile of the NOACs in Supplemental Table 2. This is not an exhaustive list of significant drug-drug interactions, and data are still lacking for many potential interactions.

Although NOACs are metabolized and excreted via a number of pathways, the P-glycoprotein pathway is of particular clinical relevance because of its inhibition by drugs commonly co-administered in AF patients.^41^ On the other hand, strong inducers of P-glycoprotein and cytochrome P-450 3A4, such as rifampicin and St John’s Wort, should be used with caution as they may result in reduced plasma levels of the NOACs.^41^

## Dose-Reduction Criteria OF NOACS for AF

The doses of NOACs must be adjusted in certain conditions when the risk of bleeding is presumably high. Table 2 shows the ABCD rule of the dose-reduction criteria of NOACs that were described in the drug labels and in major AF guidelines.^12^ For rivaroxaban, apixaban, and edoxaban, the dose-reduction criteria were adopted in their clinical trials, respectively.^36^, ^37^, ^38^ In a recent meta-analysis of these 3 trials, patients eligible for reduced-dose NOACs were at elevated risk of thromboembolic and hemorrhagic complications when treated with anticoagulants.^42^ NOACs, when appropriately dose-adjusted, had an improved benefit-harm profile compared with warfarin. This finding highlights the importance of prescribing reduced-dose NOACs for indicated patient.^42^ There was no dose-reduction criterion for dabigatran in the RE-LY (Randomized Evaluation of Long-Term Anticoagulant Therapy) trial, and its dose reduction criteria were obtained from the subanalyses of the RE-LY trial.^43^ Off-label dosing of NOACs may resulted in unfavorable outcomes.^44^

**Table 2:** ABCD Rule for Dose-Reduction of NOACs

	Dabigatran (Either 1 of the Following)	Rivaroxaban	Apixaban (≥2 of the Following)	Edoxaban (≥1 of the Following)
**Age** (≥80 y)	yes		yes	
**B**ody weight (≤60 kg)	yes		yes	yes
**C**reatinine or **Cr**Cl or eGFR	yes (eGFR 30-49 mL/min)	yes (eGFR 30-49 mL/min)	yes (Creatinine ≥1.5 mg/dL)	yes (CrCl 30-50 mL/min)
**D**rug				yes (Potent P-gp inhibitors)

Reduced doses are 110 mg (dabigatran), 15 mg (rivaroxaban), 2.5 mg (apixaban), and 30 mg (edoxaban)

CrCl = creatinine clearance; eGFR = estimated glomerular filtration rate; P-gp = p-glycoprotein.

### Consensus statement

• The ABCD rule for dose reduction of NOACs should be followed to obtain best efficacy and safety results in patients with AF.

## Major NOAC Trials

**Figure 3:**
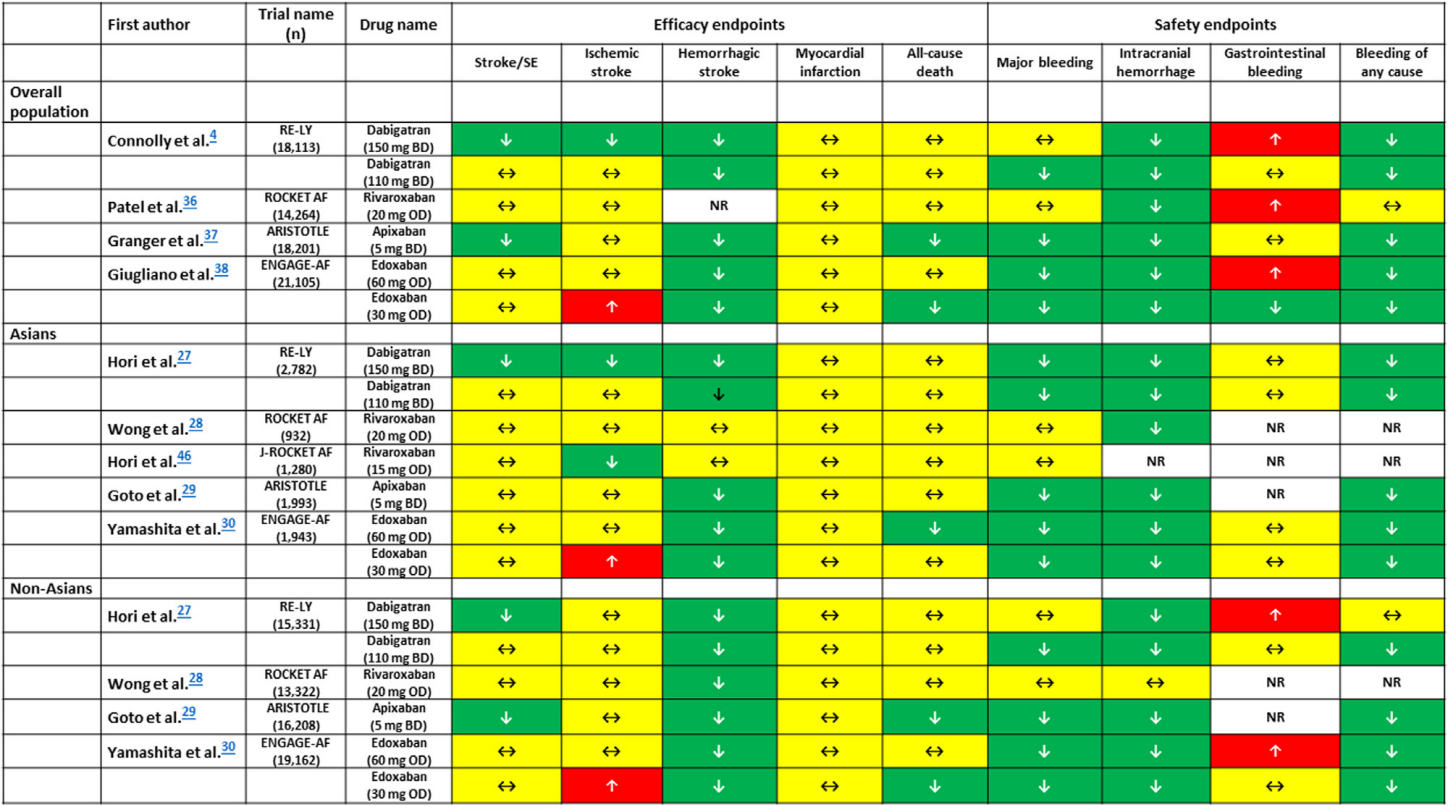
Efficacy and Safety of NOACs in Randomized Controlled Trials The efficacy and safety of non-vitamin K oral anticoagulants (NOACs) in overall, Asian, and non-Asian patients in randomized controlled trials. The **green boxes (↓)**, **yellow boxes (↔)**, and the **red boxes (↑)** indicate a decreased risk, a neutral effect, and an increased risk compared with warfarin, respectively. The **empty box** means that data have not been reported. ARISTOTLE = Apixaban for Reduction in Stroke and Other Thromboembolic Events in Atrial Fibrillation; BD = twice daily; ENGAGE-AF = Effective Anticoagulation with Factor Xa Next Generation in Atrial Fibrillation; J-ROCKET AF = Japanese Rivaroxaban Once Daily Oral Direct Factor Xa Inhibition Compared with Vitamin K Antagonism for Prevention of Stroke and Embolism Trial in Atrial Fibrillation; NR = not reported; OD = once daily; RE-LY = Randomized Evaluation of Long-Term Anticoagulation Therapy; ROCKET AF = Rivaroxaban Once Daily Oral Direct Factor Xa Inhibition Compared with Vitamin K Antagonism for Prevention of Stroke and Embolism Trial in Atrial Fibrillation; SE = systemic embolization.

The efficacy and safety of NOACs have been confirmed in 4 major clinical trials, and also by a recent patient-level meta-analysis.^4^^,^^36^, ^37^, ^38^^,^^45^ The J-ROCKET trial was the only NOAC trial dedicated specifically to Asian patients, but is underpowered for the efficacy outcomes.^46^ Figure 3 shows efficacy and safety of each NOAC in overall population, Asians, and non-Asians, respectively.

### Meta-analysis of NOAC trials

Given that the sample size of the Asian population in these clinical trials is relatively small, meta-analysis seems a good way to examine the efficacy and safety of NOACs in Asians vs non-Asians. A comprehensive meta-analysis comparing Asians versus non-Asians was performed by Wang et al^39^ (Table 3). Asians obtained greater relative risk reduction in primary efficacy endpoints and hemorrhagic stroke than non-Asians did, with *P* values for interaction of 0.045 and 0.046, respectively. More importantly, Asians acquired greater relative risk reduction in primary safety endpoints and gastrointestinal bleeding than non-Asians did, with *P* values for interaction of 0.004 and 0.041, respectively. These numbers suggested that NOACs should be preferentially indicated in Asians for stroke prevention in AF.

**Table 3:** Meta-Analysis of Standard-Dose NOACs vs Warfarin in RCTs^39^

	Asians (n = 8,928)	Non-Asians (n = 64,033)	*P* Value for Interaction
Efficacy endpoints			
Stroke/SE	0.65 (0.52-0.83)	0.85 (0.77-0.93)	0.045
Ischemic stroke	0.89 (0.87-1.17)	0.95 (0.84-1.06)	0.673
Hemorrhagic stroke	0.32 (0.19-0.52)	0.56 (0.44-0.70)	0.046
Myocardial infarction	0.97 (0.59-1.58)	0.98 (0.82-1.12)	0.977
All-cause death	0.80 (0.65-0.98)	0.91 (0.86-0.97)	0.219
Safety endpoints			
Major bleeding	0.57 (0.44-0.74)	0.89 (0.76-1.04)	0.004
Intracranial hemorrhage	0.33 (0.22-0.50)	0.52 (0.42-0.64)	0.059
Gastrointestinal bleeding	0.79 (0.48-1.31)	1.44 (1.12-1.85)	0.041

Values are HR (95% CI).

NOAC = non-vitamin K antagonist oral anticoagulant; RCT = randomized controlled trial; SE = systemic embolization.

### Consensus statement

• For Asian patients, NOACs are more effective and safer than warfarin in stroke prevention for AF.

## NOACS for Asian Patients

There was no head-to-head comparison among 4 different NOACs. Among the overall population in the 4 NOAC trials, dabigatran 150 mg twice daily and apixaban 5 mg twice daily had greater efficacy than warfarin for the primary efficacy endpoint, whereas dabigatran 110 mg twice daily, apixaban 5 mg twice daily, edoxaban 60 mg once daily, and edoxaban 30 mg once daily are superior than warfarin for the primary safety endpoint (Figure 3). These data suggest that rivaroxaban 20 mg once daily shares non-inferiority to warfarin in both efficacy and safety, whereas other NOACs are better than warfarin either in efficacy or in safety (Figure 3). Among the Asian subgroup analysis, similar findings were observed (Figure 3). When compared with warfarin, rivaroxaban 20 mg once daily in Asians (or rivaroxaban 15 mg once daily in Japanese) have similar effects in major bleeding and in hemorrhagic stroke. Some real-world evidence from Asian patients has similar findings.^47^ Considering Asian patients have higher risk of bleeding and hemorrhagic stroke, rivaroxaban 20 mg may not be the best initial choice for Asian patients.^10^

Why rivaroxaban seems to be inferior to other NOACs is not completely understood. When the sponsor planned the ROCKET AF trial, there was no phase II dose ranging trial for rivaroxaban in AF. The dose of rivaroxaban undertaken in the ROCKET AF trial (20 mg once daily) was empirically chosen, based on its dose used in the EINSTEIN VTE trial, whereas the doses used in the RE-LY, ARISTOTLE, and ENGAGE-AF trials were supported by their individual phase II dose ranging trials.^4^^,^^37^^,^^38^^,^^48^ There are major differences in the baseline characteristics of AF patients vs patients with venous thromboembolism in trials, such as the age (72 years vs 56 years), comorbidities (higher percentages vs lower percentages), and mean follow-up period (2 years vs 6 months). Therefore, the dose of rivaroxaban used in the ROCKET AF trial may be inappropriate. Interestingly, the sponsor used different dosing in most of trials after the disadvantages found in the ROCKET AF trial, such as in the PIONEER-AF PCI trial, the COMMANDER HF trial, et cetera.^49^^,^^50^ It is possible that the dosing problem caused higher bleeding events.

### Consensus statement

• For Asian patients with AF, we recommend label-adherent dabigatran, apixaban, and edoxaban as initial NOAC choices.

## NOACS in Patients with Coronary Artery Disease

Management of patients with AF and coronary artery disease is a clinical conundrum. OACs are required for the prevention of thromboembolic events, whereas antiplatelet therapy is required to prevent future atherosclerotic events. Adding single antiplatelet therapy to OAC increased risk of major bleeding by 60% to 70%, whereas dual antiplatelet therapy (DAPT) increased the risk by 130%.^51^ The clinical dilemma becomes even more complicated in patients with acute coronary syndrome (ACS) and/or percutaneous coronary intervention (PCI) when DAPT is required to prevent coronary thrombotic complications. Bleeding after PCI is associated with increased morbidity and mortality.^52^ Therefore, antithrombotic strategy should be defined to decrease the risk of bleeding while maintaining efficacy among patients with AF treated with PCI. When both bleeding risk and ischemic risk are high, bleeding risk is more impactful on clinical outcomes.^53^

### Patients with ACS undergoing PCI

There are 4 trials dedicated for patients with AF undergoing PCI (Table 4). The PIONEER AF-PCI trial was the only trial in that the doses of rivaroxaban (2.5 mg twice daily and 15 mg once daily) used in the trial have not been proven to be effective in AF patients for stroke prevention.^36^^,^^54^ The AUGUSTUS trial is the largest one, the only one testing aspirin in a placebo-controlled fashion, and the only trial that showed lower bleeding with the NOAC (apixaban) vs VKA in a direct comparison using a factorial design.^49^ The AUGUSTUS trial also enrolled patients with chronic coronary syndrome who received medical therapy alone.^49^ These patients were excluded from other trials. These 4 trials focused on bleeding as the primary endpoint, with coronary events and stroke as important secondary endpoints. To summarize, these trials showed that dual therapy with a NOAC plus a P2Y_12_ inhibitor reduced bleeding risk compared to triple therapy of warfarin, aspirin, and a P2Y_12_ inhibitor. The reduction in the bleeding risk appeared to be driven by both receiving a NOAC instead of warfarin as well as by omitting aspirin.^41^ The benefit was also observed in patients with ACS treated with medical therapy.^55^

**Table 4:** NOAC Trials for Patients With Atrial Fibrillation Undergoing PCI

First Author	Trial Name (N)	NOAC	Target Patients	Intervention Group	Control Group	Primary Endpoint	Results
Gibson et al^54^	PIONEER AF-PCI (2,124)	Rivaroxaban 2.5 mg BD Rivaroxaban 15 mg OD	ACS/PCI CCS/PCI	NOAC (2.5 mg BD) plus DAPT for 12 mo NOAC (15 mg OD) plus SAPT for 12 mo	Warfarin plus DAPT for 1, 6, 12 mo	Clinically relevant bleeding at 12 mo	Rivaroxaban 2.5 mg BD (HR: 0.63, 95% CI: 0.50-0.80) *P* < 0.001 for superiority Rivaroxaban 15 mg OD (HR: 0.59, 95% CI: 0.47-0.76) *P* < 0.001 for superiority
Cannon et al^89^	RE-DUAL PCI (2,725)	Dabigatran 110 mg BD Dabigatran 150 mg BD	ACS/PCI CCS/PCI	NOAC plus SAPT for 12 mo	Warfarin plus DAPT for 1 (BMS) or 3 (DES) mo	Major or CRNM bleeding for 14 mo	Dabigatran 110 BD (HR: 0.52, 95% CI: 0.42-0.63) *P* < 0.001 for superiority Dabigatran 150 BD (HR: 0.72, 95% CI: 0.58-0.88) *P* = 0.002 for superiority
Lopes et al^49^	AUGUSTUS (4,614)	Apixaban 5 mg BD	ACS/PCI CCS/PCI ACS/medical	NOAC or warfarin plus SAPT for 6 mo	NOAC or warfarin plus DAPT for 6 mo	Major or CRNM bleeding at 6 mo	(HR: 0.69, 95% CI: 0.58-0.81) *P* < 0.001 for superiority
Vranckx et al^90^	ENTRUST-AF PCI (1,506)	Edoxaban 60 mg OD	ACS/PCI CCS/PCI	NOAC plus SAPT for 12 mo	Warfarin plus DAPT for 12 mo	Major or CRNM bleeding at 12 mo	(HR: 0.83, 95% CI: 0.65-1.05) *P* = 0.001 for noninferiority *P* = 0.1145 for superiority

ACS = acute coronary syndrome; AUGUSTUS = Apixaban vs. Vitamin K Antagonist and Aspirin vs. Aspirin Placebo in Patients with Atrial Fibrillation and Acute Coronary Syndrome and/or Percutaneous Coronary Intervention; BD = twice daily; BMS = bare metal stent; CCS = chronic coronary syndrome; CRNM = clinical relevant non-major; DAPT = dual antiplatelet therapy; DES = drug-eluting stent; ENTRUST-AF-PCI = Edoxaban Treatment Versus Vitamin K Antagonist in Patients With Atrial Fibrillation Undergoing Percutaneous Coronary Intervention; OD = once daily; PCI = percutaneous coronary intervention; PIONEER AF-PCI = Open-Label, Randomized, Controlled, Multicenter Study Exploring Two Treatment Strategies of Rivaroxaban and a Dose-Adjusted Oral Vitamin K Antagonist Treatment Strategy in Subjects with Atrial Fibrillation who Undergo Percutaneous Coronary Intervention; RE-DUAL PCI = Randomized Evaluation of Dual Antithrombotic Therapy with Dabigatran versus Triple Therapy with Warfarin in Patients with Nonvalvular Atrial Fibrillation Undergoing Percutaneous Coronary Intervention; SAPT = single antiplatelet therapy; other abbreviation as in Table 3.

**Figure 4:**
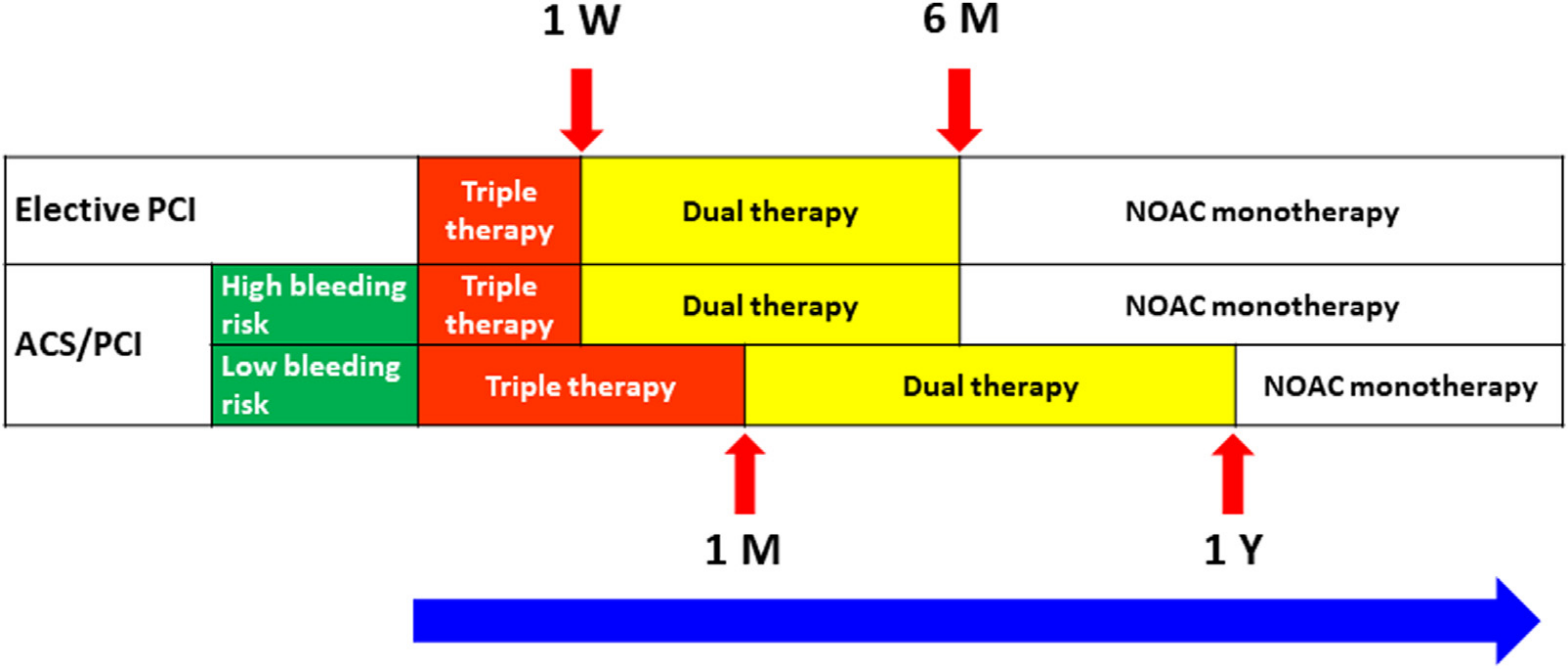
Flow Chart for AF Patients With Coronary Artery Disease/PCI For AF patients with acute coronary syndrome/percutaneous coronary interventions (PCIs), the duration of triple therapy can be reasonably shortened to 1 week if the bleeding risk is high. For patients with elective PCIs, the default duration of triple therapy is 1 week because the thrombosis risk is not high. ACS = acute coronary syndrome; M = month; W = week; other abbreviations as in Figures 1 and 3.

One should be reminded that all 4 trials were underpowered for thrombotic coronary event analyses; however, NOAC-based dual therapy seems to be safe in terms of coronary ischemic events. Several recent meta-analyses including the 4 NOAC trials have shown that there might be small but significant increases in the risk of coronary events and stent thrombosis when omitting aspirin.^56^^,^^57^ We recommend the use of aspirin during the peri-PCI period up to 1 week after PCI in patients with high bleeding risk (HAS-BLED score ≥3) (Figure 4). Given the irreversible inhibition with aspirin on platelets, residual inhibition may persist for the lifespan of platelets (7 to 10 days).^58^ For patients with low bleeding risk (HAS-BLED score <3), it is reasonable to continue aspirin for up to 1 month after PCI (Figure 4) as the thrombosis risk is highest in the first month after ACS.^59^ In the AUGUSTUS trial, the use as aspirin immediately and for up to 30 days resulted in an equal tradeoff between an increase in severe bleeding and a reduction in severe ischemic events.^60^ Extending aspirin therapy beyond 1 month after PCI is not recommended. The dual therapy consisting of a NOAC plus a P2Y_12_ inhibitor should continue after the triple therapy, and persists for 6 to 12 months, depending on the bleeding risk (Figure 4). The management strategy 1 year after PCI should be NOAC alone (Figure 4), which is a plan supported by the Japanese AFIRE trial (Atrial Fibrillation and Ischemic Events with Rivaroxaban in Patients with Stable Coronary Artery Disease) trial.^61^

Clopidogrel is the P2Y_12_ inhibitor of choice as it was used in most (88%) patients enrolled in the 4 trials. The number of patients in the 4 trials who used prasugrel was very limited (1.3% in PIONEER AF-PCI, 1.1% in AUGUSTUS, 0.5% in ENTRUST-AF PCI, and excluded in RE-DUAL PCI). In a small observational study, triple therapy of warfarin, aspirin, and prasugrel was associated with a 4-fold higher rate of bleeding.^62^ Therefore, prasugrel should not be used in AF patients undergoing PCI. Data for ticagrelor were also limited (4.3% in PIONEER AF-PCI, 12.0% in RE-DUAL PCI, 6.2% in AUGUSTUS, and 7.0% in ENTRUST-AF PCI). The bleeding risk with ticagrelor was higher than clopidogrel, and its used should be limited to patients with high thrombotic risk, such as in patients with ACS and complex PCI. Aspirin should be discontinued after the peri-PCI period when ticagrelor is combined with NOAC.^63^

### Patients undergoing elective PCI

Approximately 40% of patients enrolled in these 4 NOAC PCI trials did not have ACS. These patients received elective PCI. Because the risk of thrombosis after elective PCI is lower than that in ACS, the duration of triple therapy should be limited to peri-PCI period. The duration for double therapy can be reasonably shortened to 6 months (Figure 4).

### Patients with AF and chronic coronary syndrome

The efficacy and safety endpoints are generally consistent in patients with and without previous myocardial infarction or coronary artery disease.^64^ In a meta-analysis of 6 trials, OAC monotherapy and OAC plus single antiplatelet treatment showed similar effectiveness, but a lower risk of bleeding was found in patients with OAC alone in patients with AF and chronic coronary syndrome.^65^ The Japanese AFIRE trial showed that continuing NOAC monotherapy beyond 1 year after a revascularization procedure in AF patients not only decreased the risk of major bleeding but also demonstrated non-inferiority for the primary composite endpoint of cardiovascular events compared with the combination of NOAC and antiplatelet therapy.^61^ It is generally accepted that most AF patients with chronic coronary syndrome should be transitioned to NOAC monotherapy without an antiplatelet agent as recommended in recent guidelines or consensus.^12^^,^^41^

### Consensus statements

• For patients with AF and ACS undergoing PCI, triple therapy with a NOAC, P2Y_12_ inhibitor (clopidogrel preferred), and aspirin should be limited to 1 week after PCI when their bleeding risk is high (HAS-BLED score ≥3), followed by double therapy (a NOAC plus clopidogrel) for 6 months, and monotherapy with NOAC alone after 6 months.
• For patients with AF and ACS undergoing PCI, triple therapy can be used up to 1 month after PCI when their bleeding risk is not high (HAS-BLED score <3), followed by double therapy (a NOAC plus clopidogrel) for 12 months, and monotherapy with NOAC alone after 12 months.
• For patients with AF and ACS who receive medical therapy, triple therapy should be limited to 1 week after PCI, followed by double therapy (a NOAC plus clopidogrel) for 6 months, and monotherapy with NOAC alone after 6 months.
• For patients with AF and chronic coronary syndrome who receive elective PCI, triple therapy should be limited to 1 week after PCI, followed by double therapy (a NOAC plus clopidogrel) for 6 months, and monotherapy with NOAC alone after 6 months.
• For patients with AF and chronic coronary syndrome after more than 1 year after PCI, monotherapy with NOAC is recommended.
• All available NOACs can be used in AF patients undergoing PCI, but the approved stroke-preventive doses of NOACs should be used (rivaroxaban 20 mg once daily, dabigatran 110/150 mg twice daily, apixaban 5 mg twice daily, and edoxaban 60 mg once daily), and dose-reduction criteria should be followed. The effect of rivaroxaban 15 mg once daily is uncertain.
• The triple therapy and the double therapy regimes do not include prasugrel, whereas the use of ticagrelor should be limited to patients with high thrombotic risk, such as in patients with ACS and complex PCI.

## NOACS in Patients with Valvular Heart Diseases

Patients with moderate to severe mitral stenosis were excluded from major NOAC trials based on the previous Framingham study which showed that these patients might have a significantly increased risk of stroke, and that NOACs might not be able to prevent it.^66^ An argument has recently proposed that the Framingham study overestimated stroke risk in patients with mitral stenosis, and applicability of NOACs in patients with moderate to severe mitral stenosis is now being tested in several clinical trials (eg, NCT04045093, NCT03926156, and NCT02832544).^67^ Current guidelines remain conservative that NOACs should not be used in this situation.^12^^,^^41^^,^^68^ As for patients with mechanical valves, NOACs are clearly contraindicated based on the findings from the RE-ALIGN (Randomized Phase II Study to Evaluate the Safety and Pharmacokinetics of Oral Dabigatran Etexilate of Patients After Heart Valve Replant) trial in which the use of dabigatran in patients with mechanical heart valves was associated with increased rates of thromboembolic and bleeding complications compared with warfarin.^69^

Patients with other valvular heart diseases were included in major NOAC trials with a total of 13,585 patients.^70^ A meta-analysis of these trials shows that high-dose NOACs are more effective than warfarin in reducing stroke/systemic embolization (HR: 0.70; 95% CI: 0.58-0.86), whereas risk of major bleeding is similar (HR: 0.93; 95% CI: 0.68-1.27).^70^ When different NOACs were compared, rivaroxaban was the only NOAC that increased major bleeding (HR: 1.56; 95% CI: 1.14-2.13) when compared with warfarin (heterogeneity *P* < 0.00001). The risk of intracranial hemorrhage was also numerically higher with rivaroxaban compared with warfarin (HR: 1.27; 95% CI: 0.58-2.78; heterogeneity *P* = 0.03 compared with other high-dose NOACs). A consensus document endorsed by several international associations also mentioned that the safety of NOACs in terms of lower risk of major bleeding or intracranial hemorrhage was consistent irrespective of status of valvular heart disease, except that significantly higher rates of major bleeding were found in patients treated with rivaroxaban compared to warfarin.^68^

Patients with bioprosthetic valves were excluded in the RE-LY (Randomized Evaluation of Long-Term Anticoagulant Therapy) and the ROCKET AF (Rivaroxaban Once Daily Direct Factor Xa Inhibitor Compared With Vitamin K Antagonist for Prevention of Stroke and Embolism Trial in Atrial Fibrillation) trials. The ARISTOTLE (Apixaban for Reduction in Stroke and Other Thromboembolic Events in Atrial Fibrillation) trial enrolled very few patients with bioprosthetic valves, and formal publication was not available. A total of 191 patients with bioprosthetic valve implantation were included in the ENGAGE-AF (Effective Anticoagulation with Factor Xa Next Generation in Atrial Fibrillation) trial (n = 131 [68.6%] mitral, n = 60 [31.4%] aortic).^71^ Compared with warfarin, patients with bioprosthetic valves treated with high-dose edoxaban had lower rates of combined ischemic endpoints (4.32%/y vs 11.07%/y; HR: 0.36; 95% CI: 0.15-0.87; *P* = 0.03) and better primary net clinical outcome (stroke/systemic embolization, major bleeding, and death; 7.53%/y vs 15.77%/y; HR: 0.46; 95% CI: 0.23-0.91; *P* = 0.03).^71^ Rivaroxaban was non-inferior to warfarin in the primary endpoints in a recent trial.^72^ This is an open-labeled trial with several limitations. The statistical methods have been changed and the actual numbers of the primary endpoints were not provided.^72^ Considering that the ENGAGE AF trial is a double-blind, double-dummy trial, it seems that data for edoxaban may be more convincing in patients with bioprosthetic valves.

The anticoagulation strategy for patients with AF who undergo transcatheter aortic valve implantation (TAVI) has been a question of debate, but has been settled recently. In a recent trial in patients undergoing TAVI who were receiving OAC for appropriate indications (95% of patients with AF), OAC alone was better than OAC plus clopidogrel in the primary bleeding endpoints (risk ratio: 0.63; 95% CI: 0.43-0.90; *P* = 0.01), whereas a secondary ischemic endpoint did not show difference (risk ratio: 0.77; 95% CI for superiority: 0.46-1.31).^73^ In another trial comparing edoxaban with VKAs in patients with AF after successful TAVI, the primary efficacy outcome was 17.3 per 100 person-years in the edoxaban group and 16.5 per 100 person-years in the VKA group (HR: 1.05; 95% CI: 0.85-1.31; *P* = 0.01 for non-inferiority).^74^ A recent consensus document from the European Society of Cardiology concluded that OAC alone is suggested in patients who have AF and an indication for OAC, unless there is a recent PCI (<3 months) indicating that dual therapy with OAC plus clopidogrel may be needed.^75^

### Consensus statements

• NOACs are contraindicated in patients with mechanical valves and moderate to severe mitral stenosis.
• For patients with other valvular heart diseases, we recommend dabigatran, apixaban, and edoxaban as initial choices.
• For patients with bioprosthetic valves, we recommend edoxaban.
• For AF patients undergoing TAVI, OAC alone is indicated unless there is a recent PCI (<3 months) indicating that dual therapy with OAC plus clopidogrel may be needed.

## NOACS in Patients with a History of Stroke/Intracranial Hemorrhage

### Oral anticoagulants after AF-related ischemic stroke

The major NOAC trials excluded patients with recent ischemic stroke within 2 to 4 weeks due to concern for intracranial hemorrhage or hemorrhagic transformation.^76^ Based on data from an Asian population, NOAC showed better outcome in lowering the risk of intracranial hemorrhage and stroke compared to warfarin in AF patients with previous history of intracranial hemorrhage or stroke.^77^

The American Heart Association/American Stroke Association 2018 guideline recommended the start of OAC 4 to 14 days after the onset of ischemic stroke.^78^ This recommendation was based on a multicenter study which showed that the best time for initiating anticoagulation treatment for secondary stroke prevention is 4 to 14 days from stroke onset.^79^ Patients treated with OAC had a better outcome compared to those without OAC or those who used low molecular weight heparin.^79^ An analysis of data from an Asian population showed that the risk for stroke/systemic embolism, major bleeding, and death were comparable whether NOACs were started within 3 days or from ≥4 days after the onset of AF-related ischemic stroke/transient ischemic attack.^80^

### Consensus statements

• NOAC can be (re)initiated 1 day following a transient ischemic attack, but this is best prolonged to 14 days following a severe stroke.
• NOACs are preferred over VKA in the secondary prevention of AF-related stroke.

### Management of acute ischemic stroke while receiving OACs

Ischemic stroke in a patient while receiving NOAC therapy was milder compared to those without OAC.^81^ The 2021 European Practical Guide recommended that thrombolytic therapy should not be given within 48 hours after the last dose of NOAC. Prolonged activated partial thromboplastin time for dabigatran or prothrombin time for factor Xa inhibitors indicated the anticoagulation effect, and thrombolytic agent should not be administered immediately after acute ischemic stroke. If NOAC plasma levels are below the lower limit of detection, thrombolysis may be proceeded.^41^ In cases when the plasma level is unavailable and last NOAC intake is more than 48 hours in patients with normal renal function, thrombolysis may proceed.^41^ If the NOAC is dabigatran and idarucizumab is available, thrombolysis can be administered in selected patients after the reversal of dabigatran. If NOACs are factor Xa inhibitors and last intake is within 24 to 48 hours in patients with normal renal function, or plasma level measured more than 4 hours after intake is <30 ng/mL, thrombolysis may be given in highly selected patients. In other situations, endovascular thrombectomy may be provided if indicated. For patients receiving VKA and INR <1.7, thrombolysis may be considered according to the neurological indication.

For patients with recurrent ischemic stroke while receiving VKA or NOAC, there was no trial evidence to suggest one NOAC over the other. Nevertheless, dabigatran 150 mg is the only NOAC that decreased both ischemic and hemorrhagic stroke compared with VKA in Asia^35^; therefore, it is reasonable to recommend it in patients with recurrent ischemic stroke while on good anticoagulation (other NOACs or time-in-therapeutic-range >65% to 70% while on VKA).^10^

### Consensus statements

• In patients with recurrent ischemic stroke while on NOACs, thrombolytic therapy should not be given within 48 hours after the last dose of NOAC.
• If the NOAC is dabigatran and idarucizumab is available, thrombolysis can be given in selected patients after the reversal of dabigatran.
• If NOACs are factor Xa inhibitors and last intake is within 24 to 48 hours in patients with normal renal function, or plasma level measured more than 4 hours after intake is <30 ng/mL, thrombolysis may be given in highly selected patients.
• For patients receiving VKA and INR <1.7, thrombolysis may be considered according to the neurological indication.
• In other situations, endovascular thrombectomy may be provided if indicated.
• For Asian AF patients, dabigatran 150 mg may be considered in patients with recurrent ischemic stroke despite on other NOACs or with good anticoagulation control with VKA.

### Management of AF patients with a history of intracranial hemorrhage

Major NOAC trials excluded patients with a history of intracranial hemorrhage.^76^ Initiation of OAC in patients with AF and a history of intracranial hemorrhage should be individualized.^12^ Modifiable factors of intracranial hemorrhage such as uncontrolled hypertension, alcoholic consumption, cigarette smoking, and concomitant antiplatelet use should be corrected. OAC may be considered 2 to 4 weeks after intracranial hemorrhage.^12^ However, the 2021 European Practical Guide suggested the initiation of NOAC 4 to 8 weeks after intracranial hemorrhage.^41^ Left atrial appendage occlusion may be considered for patients with irreversible cause of intracranial hemorrhage or nonmodifiable risk factors.^12^ NOACs are preferred over VKA because of the lower risk of intracranial hemorrhage.

### Consensus statements

• In patients with a history of intracranial hemorrhage, initiation of OAC should be individualized based on the benefit of preventing stroke and the risk of recurrent intracranial hemorrhage.
• NOACs should be the preferred option because there is a lower risk of intracranial hemorrhage.
• NOAC may be started 2 weeks after intracranial hemorrhage in patients with high risk for ischemic stroke and low risk for recurrent intracranial hemorrhage.
• Left atrial appendage occlusion may be considered in patients with recurrent intracranial hemorrhage under NOACs or are contraindicated for NOACs.

## NOACS in Elderly Patients

Clinical guidelines recommended NOACs for stroke prevention in elderly patients.^11^^,^^12^ In the landmark trials on NOACs, the proportions of elderly AF patients who were ≥75 years of age ranged from 31% to 43%.^4^^,^^36^, ^37^, ^38^ A meta-analysis showed no interaction by different age groups in both efficacy and safety of NOACs.^76^ When the results between Asian and non-Asian patients were compared, standard-dose NOACs showed higher efficacy and safety relative to warfarin in Asians than in non-Asians, whereas low-dose NOACs showed similar efficacy and safety in both populations.^39^

There have been no randomized studies specifically comparing the safety between one NOAC over the other in the elderly. In the J-ROCKET AF (Japanese Rivaroxaban Once Daily Oral Direct Factor Xa Inhibition Compared with Vitamin K Antagonism for Prevention of Stroke and Embolism Trial in Atrial Fibrillation) study, the principal safety endpoints (major bleeding plus nonmajor clinically relevant bleeding) in the rivaroxaban arm were significantly increased in the elderly (age ≥75 years) (HR: 1.49; 95% CI: 1.02-2.16) but not in the non-elderly (age <75 years) (HR: 0.89; 95% CI: 0.64-1.23) when compared with the warfarin arm (*P* for interaction = 0.04).^82^ Similar results were found in the ROCKET AF trial.^83^ It may be reasonable to select other NOACs as initial choices for elderly patients in Asia.

Several studies from real-world evidence for elderly patients have been reported from Asia. A retrospective study using the Korean Health Insurance Database for elderly AF patients aged ≥80 years reported that, compared to warfarin, NOACs were associated with lower risks of ischemic stroke and were a composite of ischemic stroke and major bleeding, and a similar risk of intracranial hemorrhage.^84^ A recent prospective ANAFIE registry enrolling Japanese elderly AF patients aged ≥75 years (N = 32,275; mean age: 81.5 years) showed a high prescription rate (92%) of OAC (25% warfarin, 67% NOACs).^85^ NOACs, when compared with warfarin, were associated with lower incidences of ischemic stroke, major bleeding, intracranial hemorrhage, and all-cause death compared with warfarin.^85^

One major issue of NOAC treatment in elderly patients is “underdosing.” The recent GARFIELD-AF (Global Anticoagulant Registry in the Field-Atrial Fibrillation) registry reported that the prevalence of NOAC-underdosing was more common among Asian countries than in non-Asian countries. More importantly, underdosing was associated with a higher mortality compared with recommended doses.^86^ In the ANAFIE registry, inappropriate low-dose NOACs were prescribed in 20% to 30% of patients aged ≥75 years, particularly in patients with high bleeding risk.^87^

The ELDERCARE-AF (Edoxaban Low-Dose for Elder Care-Atrial Fibrillation) trial compared edoxaban 15 mg once daily vs placebo among very elderly (≥80 years) Japanese nonvalvular AF patients who were deemed ineligible for standard OACs due to high-bleeding risks (creatinine clearance, 15 to 30 mL/min; history of bleeding from critical organs; body weight ≤45 kg; and continuous use of nonsteroidal anti-inflammatory drugs or antiplatelet drugs).^88^ Edoxaban was superior to placebo in preventing stroke/systemic embolism (HR: 0.34; 95% CI: 0.19-0.61; *P* < 0.001) with a nonsignificant increase in major bleeding compared with placebo (HR: 1.87; 95% CI: 0.90-3.89; *P* = 0.09) and a substantial increase in gastrointestinal bleeding with edoxaban (HR: 2.85; 95% CI: 1.03-7.88). Accordingly, edoxaban 15 mg has recently been approved in Japan for very elderly AF patients who were considered ineligible for standard OAC therapy because of high bleeding risk.

### Consensus statements

• Asian real-world evidence has indicated that NOACs are preferable to warfarin even in elderly patients.
• For elderly AF patients, we recommend dabigatran 110 mg, apixaban, and edoxaban as initial choices.
• Elderly patients are often prescribed underdosed NOACs that might increase clinical events. Therefore, even in elderly patients, on-label doses of NOAC should be prioritized for stroke prevention.
• Edoxaban 15 mg may be a possible choice when on-label doses are ineligible or no OAC use is considered in elderly fragile patients at high bleeding risk.

## Future Perspectives

Previous NOAC trials excluded patients with several clinical conditions, such as rheumatic mitral stenosis, end-stage renal disease, intracranial hemorrhage, et cetera. Several ongoing trials are testing NOACs in these and other clinical settings, different non-OAC drugs, and new OACs (Table 5). The upcoming part 2 of this statement will include other important topics, such as patients with chronic kidney disease or liver disease, history of gastrointestinal bleeding, planned invasive procedure or surgery, and planned cardioversion. It will also mention how to manage bleeding events, non-pharmacological management to prevent stroke, mobile technology, and special consideration during the COVID-19 disease pandemic. It will end with prioritization of NOACs in different clinical conditions.

**Table 5:** Ongoing NOAC Trials

Clinical Conditions or Drugs	Trial Names (NCT)
Rheumatic mitral stenosis	INVICTUS (NCT02832544), DAVID-MS (NCT04045093)
Covert stroke and cognitive decline	BRAIN-AF (NCT02387229)
Early vs late strategy after ischemic stroke	TIMING (NCT02961348), ELAN (NCT03148457), OPTIMAS (NCT03759938)
Intracranial hemorrhage	ASPIRE (NCT03907046), ENRICH-AF (NCT03950076), PRESTIGE-AF (NCT03996772)
Embolic stroke of undetermined source	ARCADIA (NCT03192215)
End-stage renal disease	AXADIA-AFNET 8 (NCT02933697), SAFE-D (NCT03987711)
Device-detected AF or AHRE	ARTESiA (NCT01938248), SILENT (NCT02004509), NOAH-AFNET 6 (NCT02618577)
After successful ablation	ODIn-AF (NCT02067182), OCEAN (NCT02168829),
Left-atrial appendage occlusion	STROKECLOSE (NCT02830152), ASAP-TOO (NCT02928497), WAVECREST2 (NCT03302494), CLOSURE (NCT03463317), Occlusion-AF (NCT03642509), OPTION (NCT03795298)
Anti-diabetic drug (Metformin)	TRIM-AF (NCT03603912)
Anti-inflammatory drug (Colchicine)	IMPROVE-PVI Pilot (NCT04160117)
XIa inhibitors	
Asundexian	OCEANIC-AF (to be assigned)
Milvexian	To be determined

AF = atrial fibrillation; AHRE = atrial high rate episode; ARCADIA = AtRial Cardiopathy and Antithrombotic Drugs In Prevention After Cryptogenic Stroke; ARTESiA = Apixaban for the Reduction of Thrombo-Embolism in Patients With Device-Detected Sub-Clinical Atrial Fibrillation; ASAP-TOO = Assessment of the WATCHMAN™ Device in Patients Unsuitable for Oral Anticoagulation; ASPIRE = Anticoagulation in ICH Survivors for Stroke Prevention and Recovery; AXADIA-AFNET 8 = Compare Apixaban and Vitamin-K Antagonists in Patients With Atrial Fibrillation (AF) and End-Stage Kidney Disease-Atrial Fibrillation Network 8; BRAIN-AF = Blinded Randomized Trial of Anticoagulation to Prevent Ischemic Stroke and Neurocognitive Impairment in AF; CLOSURE = Left Atrial Appendage CLOSURE in Patients With Atrial Fibrillation Compared to Medical Therapy; DAVID-MS = Dabigatran for Mitral Stenosis Atrial Fibrillation; ELAN = Early Versus Late Initiation of Direct Oral Anticoagulants in Post-ischemic Stroke Patients With Atrial fibrillatioN; ENRICH-AF = EdoxabaN foR IntraCranial Hemorrhage Survivors With Atrial Fibrillation; IMPROVE-PVI Pilot = Impact of Short-course Colchicine Versus Placebo After Pulmonary Vein Isolation; INVICTUS = INVestIgation of rheumatiC AF Treatment Using Vitamin K Antagonists, Rivaroxaban or Aspirin Studies, Non-Inferiority; NCT = National Clinical Trial identifier; NOAH-AFNET 6 = Non-vitamin K Antagonist Oral Anticoagulants in Patients With Atrial High Rate Episodes-Atrial Fibrillation Network 6; OCEAN = Optimal Anticoagulation for Higher Risk Patients Post-Catheter Ablation for Atrial Fibrillation Trial; OCEANIC-AF = Oral faCtor Eleven A iNhibitor asundexIan as novel antithrombotiC - Atrial Fibrillation study; Occlusion-AF = Left Atrial Appendage Occlusion Versus Novel Oral Anticoagulation for Stroke Prevention in Atrial Fibrillation; ODIn-AF = Prevention of Silent Cerebral Thromboembolism by Oral Anticoagulation With Dabigatran After Pulmonary Vein Isolation for Atrial Fibrillation; OPTION = Comparison of Anticoagulation With Left Atrial Appendage Closure After AF Ablation; OPTIMAS = OPtimal TIMing of Anticoagulation After Acute Ischaemic Stroke; PRESTIGE-AF = PREvention of STroke in Intracerebral haemorrhaGE Survivors With Atrial Fibrillation; SAFE-D = Strategies for the Management of Atrial Fibrillation in patiEnts Receiving Dialysis; SILENT = Subclinical AtrIal FibrilLation and StrokE PreveNtion Trial; STROKECLOSE = Prevention of Stroke by Left Atrial Appendage Closure in Atrial Fibrillation Patients After Intracerebral Hemorrhage; TIMING = TIMING of Oral Anticoagulant Therapy in Acute Ischemic Stroke With Atrial Fibrillation; TRIM-AF = Targeting Risk Interventions and Metformin for Atrial Fibrillation; WAVECREST2 = WAveCrest Vs. Watchman TranssEptal LAA Closure to REduce AF-Mediated STroke 2; XIa = activated factor XI; other abbreviation as in Table 3.

## Funding Support and Author Disclosures

This work was supported, in part, by grants from the Ministry of Health and Welfare (MOHW111-TDU-B-211-134001), and intramural grants from the Taipei Veterans General Hospital (V111C-194). Dr Chiang has received honoraria from AstraZeneca, Boehringer Ingelheim, Daiichi-Sankyo, MSD, Novartis, Pfizer, and Sanofi. Dr Chao has received honoraria for lectures from Boehringer Ingelheim, Bayer, Pfizer, and Daiichi Sankyo. Dr Choi has received research grants or speaking fees from Bayer, BMS/Pfizer, Biosense Webster, Daiichi-Sankyo, and Medtronic. Dr Krittayaphong has received honoraria from Bayer, Boehringer Ingelheim, Daiichi-Sankyo, and Pfizer. Dr Li has received honoraria from Bayer and Boehringer Ingelheim. Dr Chen has received honoraria from Biosense Webster, St Jude Medical, Medtronic, Bayer, and Boehringer Ingelheim. Dr Okumura has received honoraria from Daiichi-Sankyo, Boehringer Ingelheim, Bristol-Myers Squibb, Medtronic, Japan lifeline, and Johnson and Johnson. Dr Lip consults and is a speaker for BMS/Pfizer, Boehringer Ingelheim, and Daiichi-Sankyo, with no fees are received personally. All other authors have reported that they have no relationships relevant to the contents of this paper to disclose.
