# Supplementary material for: Stroke Prevention in Atrial Fibrillation: A Scientific Statement of JACC: Asia (Part 1)
Source: JACC Asia. 2022 Aug 16;2(4):395–411. doi: 10.1016/j.jacasi.2022.05.005 (PMC9627863; doi:10.1016/j.jacasi.2022.05.005)
Supplement: Supplemental Data [file mmc1.docx]

**Stroke Prevention in Atrial Fibrillation: A Scientific Statement of JACC: Asia (Part 1)**

**Running title:** Stroke prevention in atrial fibrillation

Chern-En Chiang, MD, PhD ^a,b,c^; Tze-Fan Chao, MD, PhD ^b,d^; Eue-Keun Choi, MD, PhD ^e^; Toon Wei Lim, MD, PhD ^f^; Rungroj Krittayaphong, MD ^g^; Mingfang Li, MD, PhD ^h^;

Minglong Chen, MD ^h^; Yutao Guo, MD^I,j^; Ken Okumura, MD, PhD^k^;

Gregory Y.H. Lip, MD ^e,g,h,j,l^

**Supplementary Content**

Supplemental Table 1. List of working group members.

Supplemental Table 2. Pharmacokinetics and common drug-drug interactions of NOAC

Supplemental Figure 1. Summary of the use of CHA_2_DS_2_-VASc and HAS-BLED scores for stroke and bleeding risk assessment/re-assessment for Asian AF patients

**Supplemental Table 1**. List of working group members.

| Names | Institutions |
| --- | --- |
| Chern-En Chiang (Chair) | Taipei Veterans General Hospital, Taipei, Taiwan |
| Tze-Fan Chao | Taipei Veterans General Hospital, Taipei, Taiwan |
| Eue-Keun Choi | Seoul National University Hospital, Seoul, Republic of Korea |
| Toon Wei Lim | National University Hospital, Singapore, Singapore |
| Rungroj Krittayaphong | Mahidol University, Bangkok, Thailand |
| Mingfang Li | First Affiliated Hospital of Nanjing Medical University, Nanjing, China |
| Minglong Chen | First Affiliated Hospital of Nanjing Medical University, Nanjing, China |
| Yutao Guo | Chinese PLA General Hospital, Beijing, China |
| Ken Okumura (Co-chair) | Saiseikai Kumamoto Hospital, Kumamoto, Japan |
| Gregory Y.H. Lip | University of Liverpool & Liverpool Heart and Chest Hospital, Liverpool, UK |

**Supplemental Table 2**. Pharmacokinetics and common drug-drug interactions of NOAC

| Drug | Dabigatran^1,2^ | Apixaban^3^ | Edoxaban^4^ | Rivaroxaban^5,6^ |
| --- | --- | --- | --- | --- |
| Bioavailability | 3-7% | 50% | 62% | 66% without food, 100% with food |
| Renal clearance | 80% | 27% | 50% | 35% |
| Plasma protein Bound | 35% | 87% | 55% | 95% |
| P-gp substrate | Yes | Yes | Yes | Yes |
| CYP3A4 substrate | No | Yes (25%) | No (<5%) | Yes (18%) |
| Time to peak levels (hours) | 3 | 3 | 2-4 | 2-4 |
| Half-life (hours) | 12-17 | 12 | 10-14 | 5-9 (young)  11-13 (old) |
| Common drug-drug interactions (non-exhaustive list) | | | | |
| Contraindicated:  Increased plasma levels | Dronedarone, protease inhibitors, ketoconazole | Ketoconazole |  | Dronedarone, protease inhibitors, ketoconazole |
| Caution, monitor, reduce dose: increased plasma levels | Amiodarone, quinidine, verapamil, ticagrelor, clarithromycin | Dronedarone, clarithromycin, naproxen | Dronedarone, quinidine, verapamil, erythromycin, ketoconazole | Verapamil, clarithromycin, fluconazole |
| Contraindicated: Reduced plasma levels | Rifampicin, St John’s wort | Rifampicin, St John’s wort | Rifampicin, St John’s wort | Rifampicin, St John’s wort |

CYP = cytochrome P-450; NOAC = non-vitamin K antagonist oral anticoagulant; P-gp = p-glycoprotein.

**Supplemental Figure 1**. Summary of the use of CHA_2_DS_2_-VASc and HAS-BLED scores for stroke and bleeding risk assessment/re-assessment for Asian AF patients.


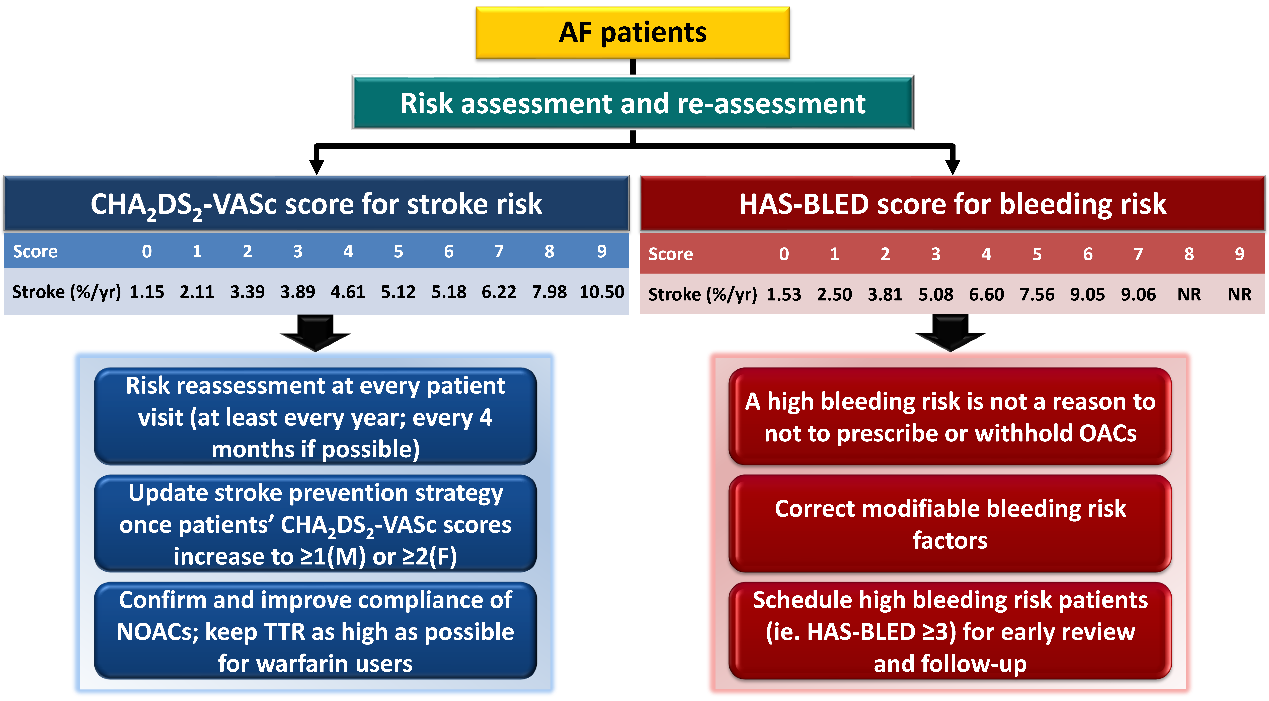


AF = atrial fibrillation; CHA_2_DS_2_-VASc = congestive heart failure, hypertension, age ≥75 (doubled), diabetes, stroke (doubled)-vascular disease, age 65-74 and sex category (female); F = female; HAS-BLED scores = hypertension, abnormal renal/liver function (1 point each), stroke, bleeding history or predisposition, labile INR, elderly (> 65 years), drugs/alcohol concomitantly (1 point each); NOACs = non-vitamin K antagonist oral anticoagulants; OACs = oral anticoagulants; TTR = time in therapeutic range; yr = year.

The figure was redrawn, and data were adapted from the papers by Chao et al.^7-10^

**References**

1. Blech S, Ebner T, Ludwig-Schwellinger E, Stangier J, Roth W. The metabolism and disposition of the oral direct thrombin inhibitor, dabigatran, in humans. *Drug Metab Dispos.* 2008;36(2):386-399.

2. Stangier J, Rathgen K, Stähle H, Gansser D, Roth W. The pharmacokinetics, pharmacodynamics and tolerability of dabigatran etexilate, a new oral direct thrombin inhibitor, in healthy male subjects. *Br J Clin Pharmacol.* 2007;64(3):292-303.

3. Raghavan N, Frost CE, Yu Z, et al. Apixaban metabolism and pharmacokinetics after oral administration to humans. *Drug Metab Dispos.* 2009;37(1):74-81.

4. Ogata K, Mendell-Harary J, Tachibana M, et al. Clinical safety, tolerability, pharmacokinetics, and pharmacodynamics of the novel factor Xa inhibitor edoxaban in healthy volunteers. *J Clin Pharmacol.* 2010;50(7):743-753.

5. Kubitza D, Becka M, Voith B, Zuehlsdorf M, Wensing G. Safety, pharmacodynamics, and pharmacokinetics of single doses of BAY 59-7939, an oral, direct factor Xa inhibitor. *Clin Pharmacol Ther.* 2005;78(4):412-421.

6. Mueck W, Stampfuss J, Kubitza D, Becka M. Clinical pharmacokinetic and pharmacodynamic profile of rivaroxaban. *Clin Pharmacokinet.* 2014;53(1):1-16.

7. Chao T-F, Liu C-J, Tuan T-C, et al. Comparisons of CHADS2 and CHA2DS2-VASc scores for stroke risk stratification in atrial fibrillation: Which scoring system should be used for Asians? *Heart Rhythm.* 2016;13(1):46-53.

8. Chao TF, Lip GYH, Lin YJ, et al. Major bleeding and intracranial hemorrhage risk prediction in patients with atrial fibrillation: Attention to modifiable bleeding risk factors or use of a bleeding risk stratification score? A nationwide cohort study. *Int J Cardiol.* 2018;254:157-161.

9. Chang TY, Lip GYH, Chen SA, Chao TF. Importance of Risk Reassessment in Patients With Atrial Fibrillation in Guidelines: Assessing Risk as a Dynamic Process. *Can J Cardiol.* 2019;35(5):611-618.

10. Chao TF, Joung B, Takahashi Y, et al. 2021 Focused Update Consensus Guidelines of the Asia Pacific Heart Rhythm Society on Stroke Prevention in Atrial Fibrillation: Executive Summary. *Thromb Haemost.* 2022;122(1):20-47.
